# Clinical spectrum and management of dystonia in patients with Japanese encephalitis: A systematic review

**DOI:** 10.1002/brb3.2496

**Published:** 2022-01-13

**Authors:** Roshan Aryal, Suraj Shrestha, Sushan Homagain, Sunit Chhetri, Kshitiz Shrestha, Sanjeev Kharel, Ragesh Karn, Reema Rajbhandari, Bikram Prasad Gajurel, Rajeev Ojha

**Affiliations:** ^1^ Department of Medicine, Maharajgunj Medical Campus Institute of Medicine Kathmandu 44600 Nepal; ^2^ Department of Medicine B.P. Koirala Institute of Health Sciences Dharan 56700 Nepal; ^3^ Department of Neurology Tribhuvan University Institute of Medicine Kathmandu 44600 Nepal

**Keywords:** dystonia, Japanese encephalitis, movement disorder, review

## Abstract

**Background:**

Japanese encephalitis (JE) is a potentially fatal viral infection with a wide range of manifestations and can also present with a variety of movement disorders (MD) including dystonia. Dystonic features in JE are uncommon. Here, we have tried to summarize the clinical features and management of dystonia among JE patients with a comprehensive literature search.

**Methods:**

Various databases, including PubMed, Embase, and Google Scholar, were searched against the predefined criteria using suitable keywords combination and boolean operations. Relevant information from observational and case studies was extracted according to the author, dystonic features, radiological changes in the brain scans, treatment options, and outcome wherever provided.

**Result:**

We identified 19 studies with a total of 1547 JE patients, the diagnosis of which was confirmed by IgM detection in serum and/or cerebrospinal fluid in the majority of the patients (88.62%). 234 (15.13%) of JE patients had dystonia with several types of focal dystonia being present in 131 (55.98%) either alone or in combination. Neuroimaging showed predominant involvement of thalami, basal ganglia, and brainstem. Oral medications including anticholinergics, GABA agonists, and benzodiazepines followed by botulinum toxin were the most common treatment modalities.

**Conclusion:**

Dystonia can be a disabling consequence of JE, and various available medical therapies can significantly improve the quality of life. Owing to insufficient studies on the assessment of dystonia associated with JE, longitudinal studies with a larger number of patients are warranted to further clarify the clinical course, treatment, and outcome of dystonia.

## Graphical abstract

Dystonia, a type of movement disorder, can also be manifested in Japanese encephalitis showing predominant involvement of thalami, basal ganglia, and brainstem. Numerous available medical therapies can improve the quality of life in dystonic patients.

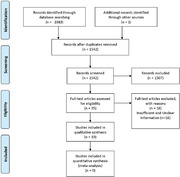

## 1 INTRODUCTION

Movement disorders (MD) can be primary, which is a presentation of an underlying neurodegenerative disorder, or secondary that arises from other disease states or brain injury (Jhunjhunwala et al., 2014). Infections, cerebrovascular disease, space‐occupying lesions, and trauma have been described as various etiological factors causing secondary MDs (SMDs) (Mehanna & Jankovic, 2013). Dystonia, a type of MD, is characterized by sustained muscle contractions producing twisting, repetitive, and patterned movements or abnormal postures (Albanese, 2003; Steeves et al., 2012). A broad variety of dystonia, such as orofacial, limb, and axial, are reported (Misra & Kalita, 2010). Dystonia has a wide clinical spectrum ranging from minimal or benign self‐limiting features to severe cases (Albanese, 2003; Fernández‐Alvarez, 2010).

Japanese encephalitis (JE), caused by the Japanese encephalitis virus (JEV) and transmitted by *Culex* mosquitoes, is the most common human endemic encephalitis (Solomon et al., 1998). It is found throughout South and Southeast Asia, encompassing an area delimited by Pakistan to the west, the Philippines and Japan to the east, and the Australian Torres Strait Islands to the south (Turtle & Solomon, 2018). The most inclusive approximation of incidence within the past decade suggests that 69,000 cases of JE occur every year (Campbell et al., 2011). Once replication completes, the virus amplifies to produce viremia and crosses the blood–brain barrier to enter the central nervous system, causing a diffuse brain infection or encephalitis in some cases (Hoke et al., 1992). The onset of the illness ranges from abrupt to gradual, and the disease progresses through prodromal (2 to 5 days), encephalitis (1 to 3 weeks), and late (weeks to several months) stages, with a variety of MD associated, including dystonia (Richter & Shimojyo, 1961; Tiroumourougane, 2002).

Netravathi et al. in their study had reported infectious causes representing up to 20.4% of all secondary MD (Netravathi et al., 2012). Viral organisms such as JEV, human immunodeficiency virus (HIV), dengue, mumps, polio, coxsackie, varicella‐zoster, and measles have been reported causing a whole range of MDs (Duvoisin & Yahr, 1965; Howard & Lees, 1987). MDs are common in JE and have been reported in up to 60% of patients (Misra & Kalita, 2010). Kalita and Misra in their study on JE had reported predominant post‐encephalitic dystonia (Kalita & Misra, 2000; Tse et al., 2004). Further, only a small proportion of studies on dystonia in JE have been conducted. In this review, we compile and describe the clinical features, diagnostic findings, treatments, and outcomes of dystonic patients with JE.

## 2 METHODOLOGY

### 2.1 Data collection

Databases such as PubMed, Embase, and Google Scholar were searched to identify all relevant published articles from 2000 until September 1, 2021, using the terms dystonia, movement disorder, muscle dystonia, dyskinesia, dystonic disorders, Japanese encephalitis, Encephalitis, Japanese B Viral Encephalitis, Viral Encephalitis, JE with suitable boolean operators “AND” and “OR” wherever deemed necessary. Further, references of included articles were screened for additional studies. The search strategy used is provided in Appendix 1. Studies obtained from the search were exported to ENDNOTE reference software version 8.0.2 (Thomson Reuters, Stamford, CT, USA) in the compatible formats. Screening of duplicate articles was done by ENDNOTE at first, and then manually and was subsequently removed.

### 2.2 Inclusion and exclusion criteria

All articles were considered eligible for inclusion if:

1. Published in English
2. Reporting on dystonia after JE
3. Human studies.

The following exclusion criteria were applied:

1. Viewpoints, conference papers, commentaries, editorials, letters
2. Research protocols, review articles
3. Not in English
4. Full text not available
5. Studies with insufficient information
6. Adverse events following JE vaccination
7. Cases described under a broad set of diseases where required details could not be secluded
8. Movement disorder in JE other than dystonia

**FIGURE 1:**
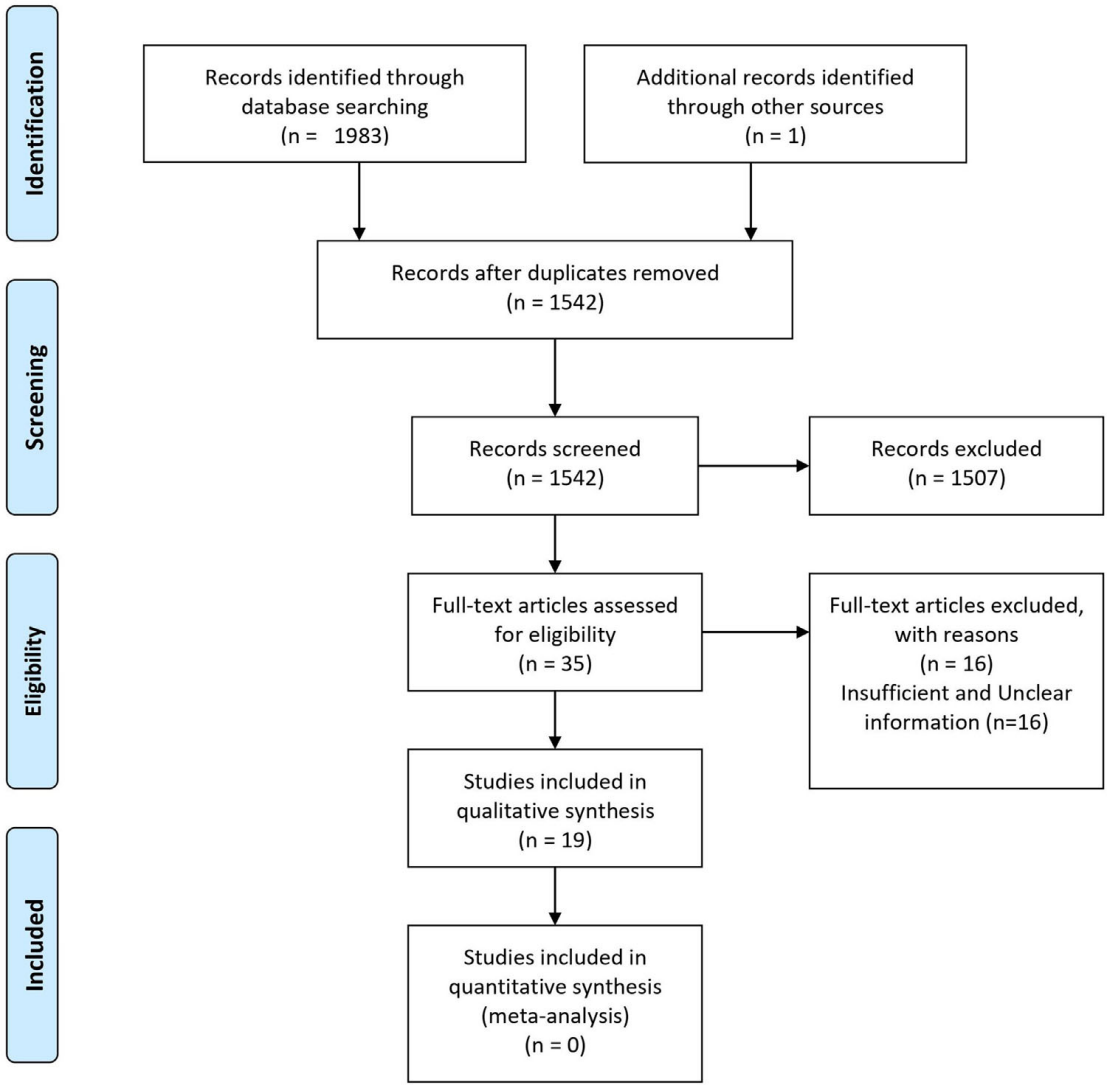
PRISMA diagram of the study identification and selection process

For two or more studies, including the same set of patients, we included the study with more sample size. The PRISMA diagram detailing the selection process is shown in Figure 1.

### 2.3 Data extraction and management

Detailed review of the selected studies was performed by the authors (RA and SS), and the following information was extracted: name of the first author, year of publication, country of study, study design, study period, sample size, number of patients with JE, sex, age groups, modality of diagnosis of JE, number of JE patients with dystonia, type of dystonia, computed tomography (CT)/magnetic resonance imaging (MRI) findings, additional diagnostic tools used (if any), methods of treatment of dystonia, and outcome. The information was recorded in Microsoft Excel version 2019 (Microsoft Corp., Redmond, WA, USA). Any disagreement was resolved by mutual consensus with the third author (SH).

## 3 RESULTS

### 3.1 Literature search results

The search returned 1983 articles and one was added after going through the references of selected papers (PubMed: 580, Embase: 1403, and Google Scholar: 1). After removing the duplicates, 1542 articles were evaluated based on their titles and abstracts, and 35 full‐text articles were reviewed using the eligibility criteria. Finally, 19 articles met our inclusion criteria and were included in the study.

### 3.2 Study characteristics

Of the 19 studies included, nine were prospective studies, five case reports, two observational studies, two retrospective analyses, and one cohort study. The total number of JE patients included was 1547, ranging from 1 to 649 per included study. Most studies were from India (12), followed by Nepal (2), and each from China, Japan, Malaysia, Vietnam, and Italy. Details about selected studies can be found in Table 1.

**TABLE 1:** Study characteristics of included studies

**Author**	**Year**	**Study period (in years)**	**Country**	**Study design**	**Sample size**	**Number of JE patients**	**Male**	**Female**	**Children (< 14yrs)**	**Adults**	**Confirmation of JE**
Basumatary et al., 2013	2013	3	India	Prospective study	148	148	94	54	44	104	JE virus‐specific IgM antibody detection in serum and CSF in 121. Clinical and neuroimaging features for the remaining 27
Dutta et al., 2021	2021	2.5	India	Prospective cohort study	194	56	32	24	56	–	Anti JEV IgM in CSF or in both serum and CSF
Ghosh et al., 2020	2020	–	India	Case report	1	1	–	1	–	1	JEV IgM by ELISA in serum
Hamano et al., 2004	2004	–	Japan	Case report	1	1	–	1	–	1	NA
Kalita & Misra, 2000	2000	7	India	Prospective study	50	50	50	0	NA	NA	Hemagglutinin inhibition titer, 2 mercaptoethanol test, IgM antibody capture in CSF, and viral isolation
Kalita et al., 2003	2003	10	India	Retrospective analysis	67	67	NA	NA	30	37	Antibody titer (hemagglutinin inhibition for JE virus or 2‐mercaptoethanol test in serum or IgM capture through ELISA), polymerase chain reaction
Kalita et al., 2011	2011	5	India	Prospective study	209	14	8	6	7	7	Anti JEV IgM through ELISA
Kalita et al., 2016	2016	10	India	Retrospective analysis	137	97	NA	NA	29	68	CSF anti‐JEV IgM using ELISA
Liao et al., 2010	2009	–	China	Case reports	3	3	3	0	–	3	JEV‐specific IgM antibodies were detected in all the patients by using an IgM antibody capture ELISA
Maurya et al., 2020	2020	–	Nepal	Case report	1	1	1	0	–	1	CSF anti‐JEV IgM using ELISA
Misra & Kalita, 2002	2002	6	India	Observational study	50	50	39	11	NA	NA	Essential criteria and at least two supportive criteria
Misra & Kalita, 2010	2010	5	India	Prospective and observational study	209	68	NA	NA	NA	NA	CSF anti‐JEV IgM using ELISA
Murgod et al., 2001	2001	2	India	Observational study	109	15	NA	NA	NA	NA	JEV specific IgM antibody using ELISA in serum and CSF
Ooi et al., 2008	2008	7	Malaysia	Cohort study	900	118	69	49	118	–	JEV IgM in CSF (All) and in 102 in serum as well
Pradhan et al., 2001	2001	1	India	Prospective and observational study	6	6	5	1	4	2	Titers of hemagglutinin inhibition antibody
Rayamajhi et al., 2006	2006	1	Nepal	Prospective and observational study	133	58	33	25	58	–	Anti JEV IgM in serum and CSF
Sarkari et al., 2012	2011	12	India	Prospective study	1282	649	NA	NA	NA	649	Anti JEV IgM in serum and CSF, and viral isolation
Solomon et al., 2002	2002	3	Vietnam	Prospective study	555	144	82	62	134	10	Anti JEV IgM in acute and convalescent sera and CSF
Spagnolo et al., 2014	2013	–	Italy	Case report	1	1	1	–	NA	NA	Clinical and liquorial findings

Abbreviations: CSF, cerebrospinal fluid; ELISA, enzyme linked immunosorbent assay; JE, Japanese encephalitis; JEV, Japanese encephalitis virus; IgM, immunoglobulin M; NA, not available.

### 3.3 Demographic and clinical characteristics

The age of the patients ranged from 1 to 78 years. Out of 1547 patients with JE, 417 were male and 234 were female, and gender information was not available for five studies comprising 896 patients. Children less than 14 years with JE were 480 in number while 833 patients were greater than 14 years, and no age‐wise information was present for 234 patients from five studies. A comprehensive summary of the demography and clinical characteristics of the patients of the included studies is given in Table 2.

**TABLE 2:** Demographic and clinical features of dystonic patients

**Author**	**Year**	**Number of JE patients**	**Number of patients with dystonia**	**Male**	**Female**	**Children**	**Adult**	**Type of dystonia**	**Onset of dystonia**	**Severity of dystonia**
Basumatary et al.	2013	148	38	NA	NA	19	19	Generalized (*n* = 30), oromandibular (*n* = 8)	NA	NA
Dutta et al.	2021	56	12	NA	NA	56	–	NA	NA	NA
Gosh et al.	2020	1	1	–	1	–	1	Upper Limb (*n* = 1)	NA	NA
Hamano et al.	2004	1	1	–	1	–	1	Laryngeal (*n* = 1)	NA	NA
Kalita et al.	2000	50	9	NA	NA	NA	NA	Axial resulting in retrocollis and opisthotonus (*n* = 5). Jaw opening dystonia (*n* = 2). Teeth clenching (*n* = 1). Oculogyric crisis and neck deviation (*n* = 1)	1–3 weeks after the fever	Markedly severe in 5 (0, normal; 1, slight; 2, moderate; 3, severe; 4, marked)
Kalita et al.	2003	67	27	NA	NA	20	7	NA	NA	NA
Kalita et al.	2011	14	14	8	6	7	7	Oromandibular (*n* = 14) associated with upper limb (*n* = 1), neck and blepharospasm (*n* = 1), neck (*n* = 2) and neck, trunk plus limb (*n* = 6)	2–4 weeks after encephalitis	Median severity score: 4 (0, none; 1, mild; 2, moderate; 3, severe; 4, markedly severe)
Kalita et al.	2016	97	30	NA	NA	NA	NA	NA	NA	NA
Liao et al.	2009	3	1	1	–	–	1	Generalized (*n* = 1)	NA	NA
Maurya et al.	2020	1	1	1	–	–	1	Oromandibular (*n* = 1)	2nd week of illness	Grade 4 (range 0 to 4)
Misra et al.	2002	50	8	NA	NA	NA	NA	Axial (*n* = 8) associated with limb (*n* = 6) and jaw (*n* = 2)	NA	NA
Misra et al.	2010	68	38	NA	NA	NA	NA	Generalized (*n* = 26). Focal (*n* = 12) (oromandibular [*n* = 5], oromandibular and neck [*n* = 6], neck only [*n* = 1])	NA	Moderate to markedly severe. Markedly severe in 14
Murgod et al.	2001	15	3	NA	NA	2	1	Generalized (*n* = 2), right hemidystonia (*n* = 1)	NA	NA
Ooi et al.	2008	118	2	NA	NA	2	–	NA	NA	NA
Pradhan et al.	2001	6	1	NA	NA	NA	NA	NA	12–32 days after recovery from first phase of illness	NA
Rayamajhi et al.	2006	58	2	NA	NA	2	–	Truncal (*n* = 2)	NA	NA
Sakari et al.	2011	649	43	NA	NA	–	43	Generalized (*n* = 43)	NA	NA
Solomon et al	2002	144	2	NA	NA	NA	NA	Mandibular (*n* = 2)	NA	NA
Spagnolo et al.	2013	1	1	1	–	NA	NA	Generalized dystonia along with cervical dystonic tremor (*n* = 1)	NA	NA

Abbreviation: NA, not available.

Dystonia was present in 234 patients, that is, 15.13% of patients with JE. Gender information was present for only 19 patients from six studies, out of which 11 were male and 8 were female. One hundred and eight dystonic patients were children, while 81 were adults with no information on age in eight studies with 45 patients.

Generalized dystonia was present in 103 (44.02%) patients. One hundred and thirty‐one (55.98%) patients had focal dystonia either alone or in combination, which included, oromandibular in 43, truncal in 24, neck in 20, limb in 17, laryngeal in 1, blepharospasm in 1, oculogyric crisis in 1, and right hemi‐dystonia in 1. Four studies consisting of 72 patients had no information about the type of dystonia. Only five studies with 52 dystonic patients mentioned the onset of dystonia. Kalita et al. mentioned the onset of dystonia as 1 to 3 weeks after the fever in a study comprising nine dystonic patients from 50 JE cases (Kalita & Misra, 2000). Another study by Kalita et al. consisted of 14 patients with dystonia, which occurred 2 to 4 weeks after encephalitis (Kalita et al., 2011). Dystonia was present 12 to 32 days after recovery from the first phase of illness in one patient, as mentioned by Pradhan et al., and Maurya et al. revealed that oromandibular dystonia in an 11‐year‐old boy was present during the second phase of the JE illness (Maurya et al., 2020; Pradhan et al., 2001).

Only four studies with 62 dystonic patients had information about the severity of dystonia. Two studies also stated the severity index as 0: normal, 1: slight, 2: moderate, 3: severe, and 4: marked. Five patients had markedly severe dystonia (MSD) in the study by Kalita et al., with grade 4 dystonia appearing over 1 to 3 weeks (Kalita & Misra, 2000). Dystonic spasms occurred in these five patients with 20 to 30 attacks per day, and the duration of each attack was 2 to 3 min. During the attacks of MSD, all the patients had grade 4 retrocollis, opisthotonos, and limb dystonia. Along with this, fever, severe pain, exhaustion, tachycardia, sweating, tachypnea, hypertension, and pupillary dilatation were also associated. Although fixed dystonia persisted, attacks of MSD were decreased during the night but were aggravated with full bladder and fever. Misra et al. also described the presence of MSD in 14 patients in whom dystonic spasms occurred every 10 to 30 min, each episode lasting 2 to 10 min, accompanying exhaustion, autonomic dysfunction, breathing, and feeding difficulty (Misra & Kalita, 2010). The median severity score of dystonia was 4 in 14 dystonic patients, as mentioned by Kalita et al. in another study (Kalita et al., 2011). Grade 4 oromandibular dystonia, with the range of severity of dystonia ranging from 0 to 4, as described by Maurya et al. in one patient, led to persistent mouth opening and tongue protrusion causing impairment in swallowing, drooling of saliva, and difficulty in speaking (Maurya et al., 2020).

### 3.4 Diagnostic findings

JE was confirmed by JEV‐specific IgM antibody detection in serum and/or cerebrospinal fluid (CSF) in 13 studies comprising 1371 patients with JE. Along with the viral‐specific IgM detection, hemagglutination inhibition titer and 2‐mercaptoethanol test were also used for the confirmation of JE in 117 patients in two studies (Kalita & Misra, 2000; Kalita et al., 2003). Titers of hemagglutinin inhibition antibodies were solely used for six patients, whereas no confirmatory test was mentioned in one study (Hamano et al., 2004; Pradhan et al., 2001). Polymerase chain reaction and viral isolation methods were also used along with the above‐mentioned investigation for 67 patients in one study and 649 patients from two studies, respectively (Kalita et al., 2003; Sarkari et al., 2012). Misra et al. used essential criteria which were patients presenting with acute encephalitis syndrome (AES) characterized by fever and altered sensorium in which malaria and septic meningitis have been excluded, along with the presence of any two of the supportive criteria: (1) coming from JE endemic area; (2) with thalamic involvement on CT or MRI; or (3) with a fourfold rise of IgG antibodies against JE virus by hemagglutination inhibition test, a positive mercaptoethanol test, Mac‐ELISA, or virus isolation in the CSF (Misra & Kalita, 2002). Summary regarding confirmation of JE in included studies is given in Table 1.

MRI brain was done in 186 patients with dystonia, and all of them had abnormal results, with the most common site of the lesion being the thalamus. Abnormal signals were also present in the basal ganglia, brainstem, cortex, and substantia nigra. Only the thalamus was affected in two patients. CT head was performed in 76 patients, all of which showed hypodensity in bilateral thalami. Single photon emission computed tomography (SPECT) was performed in 15 patients, which showed areas of hypoperfusion in the thalamus, basal ganglia, and cortex in 14 patients and asymmetric decrease in striatal uptake in one patient. No information was present about the radiological investigations in three studies comprising 93 dystonic patients as shown in Table 3.

**TABLE 3:** Radiological investigations with findings, treatment, and outcome in dystonic patients with JE

**Author**	**Year**	**Number of JE patients**	**Number of patients with dystonia**	**Radiological investigations**	**Radiological findings**	**Treatment received for dystonia**	**Duration of treatment**	**Outcome defined as**	**Outcome**
Basumatary et al.	2013	148	38	CT, MRI	Hyperintense in T2 and FLAIR, and isointense to slightly hypointense in T1 in thalamus, basal ganglia, midbrain and pons	NA	NA	NA	Improved in all the children
Dutta et al.	2021	56	12	MRI	Thalamic involvement, basal ganglia, cortex, brainstem, medial temporal lobe	NA	NA	NA	Improved in 6–9 months
Gosh et al.	2020	1	1	MRI	Asymmetrical (right > left) bilateral thalamic and midbrain lesions, hyperintense in T2 and FLAIR and mild diffusion restriction in DWI	Trihexyphenidyl and clonazepam	NA	NA	Dystonia improved at 6 months F/U
Hamano et al.	2004	1	1	MRI	High intensity in the globus pallidus and thalamus on FLAIR	Tracheostomy	NA	NA	Stridor improved
Kalita et al.	2000	50	9	CT, MRI	CT showed bilateral low density in thalami. Hypointense in T1‐weighted sequence and hyperintense in T2‐weighted sequence in bilateral thalamus, basal ganglia and midbrain.	6–24 mg trihexyphenidyl, 15–30 mg baclofen, 15–30 mg diazepam, 25–100 mg tetrabenazine, and 1.0–2.0 mg haloperidol in various combinations	NA	NA	Subsided in 6 months
Kalita et al.	2003	67	27	CT, MRI	Abnormal signals in bilateral thalamus, basal ganglia, pons, and cortex.	NA		At 6 months, poor (bedridden), partial (needing help with daily activities) and complete (able to perform activities independently) recovery	NA for dystonia
Kalita et al.	2011	14	14	MRI, SPECT	MRI: Abnormal signals in bilateral thalamus, basal ganglia and brainstem, cortex. SPECT: Areas of hypo and hyper perfusion.	Multiple doses of trihexyphenidyl, baclofen, diazepam, tetrabenazine, and haloperidol in various combinations	NA	At 6 months on the basis of activities of daily living into poor, partial and complete recovery	Complete recovery in 2.
Kalita et al.	2016	97	30	MRI	Thalamic and basal ganglia involvement	NA	NA	NA	NA
Liao et al.	2009	3	1	MRI, SPECT	Specific to JE not available	NA	NA	NA	NA
Maurya et al.	2020	1	1	MRI	Hyperintensities in bilateral thalami, caudate, globus pallidus, right substantia nigra, parietal lobe on T2 and FLAIR	Oral: Sodium valproate, tetrabenazine, trihexyphenidyl, and clonazepam. Botulinum toxin injection 40 units in bilateral genioglossus and lateral pterygoid (10 units each)	Oral for 2 weeks; when not improved, inj. botulinum toxin was used	NA	Significant reduction in dystonia from grade 4 to 2 in 3 months follow‐up
Misra et al.	2002	50	8	MRI	Hyperintense lesion in bilateral thalamus and basal ganglia	NA	NA	At 3 months, poor, partial, and complete	Poor: 4; partial: 0; complete: 4
Misra et al.	2010	68	38	MRI	Lesions on bilateral thalami and substantia nigra	Varying combination of trihexyphenidyl, diazepam, clonazepam, haloperidol, baclofen, tetrabenazine	NA	At 6 months, poor (bedridden), partial (dependent for activities of daily living), and complete (independent for activities of daily living) recovery	Disappeared in 71% at 6 months
Murgod et al.	2001	15	3	MRI	Lesions on bilateral thalami, substantia nigra	NA	NA	NA	NA
Ooi et al.	2008	118	2	NA	NA	NA	NA	NA	NA
Pradhan et al.	2001	6	1	MRI	Involvement of thalamus, midbrain tegmentum, substantia nigra, basal ganglia, and cerebral cortex	NA	NA	NA	NA
Rayamajhi et al.	2006	58	2	CT	Bilateral hypodensity in thalamus	NA	NA	NA	NA
Sakari et al.	2011	649	43	NA	NA	NA	NA	NA	NA
Solomon et al	2002	144	2	NA	NA	NA	NA	NA	NA
Spagnolo et al.	2013	1	1	MRI	Hypointense thalamic lesion in T1 and T2	Botulinum toxin injection, anticholinergics	NA	NA	Improved over weeks to months

Abbreviations: CT, computed tomography; DWI, diffusion‐weighted imaging; FLAIR, fluid‐attenuated inversion recovery; MRI, magnetic resonance imaging; NA, not available; SPECT, single photon emission computed tomography.

### 3.5 Treatment for dystonia

Only seven studies mentioned the treatment of dystonia associated with JE, as shown in Table 3. Oral medications were the most common method for the treatment used in six studies. Trihexyphenidyl, diazepam, clonazepam, haloperidol, baclofen, and tetrabenazine (TBZ) in various combinations were used for treating dystonia in three studies that consisted of 61 patients (Kalita & Misra, 2000; Kalita et al., 2011; Misra & Kalita, 2010). Out of these three studies, the study by Kalita et al. comprising five dystonic patients was the only one to mention the dosage of the used oral medications, where 6–24 mg trihexyphenidyl, 15–30 mg baclofen, 15–30 mg diazepam, 25–100 mg TBZ and 1.0–2.0 mg haloperidol in several combinations which led to initial response followed by worsening of dystonia and reintroduction of the same combination after transient termination resulted in some relief (Kalita & Misra, 2000). These five patients also had dystonic spasms, and during severe spasms, injection of haloperidol and diazepam were used. Trihexyphenidyl with clonazepam was used for upper limb dystonia in one patient (Ghosh et al., 2020). Maurya et al. mentioned the use of oral sodium valproate, TBZ, trihexyphenidyl, and clonazepam for oromandibular dystonia in one patient for 2 weeks, and when there was no improvement, 40 units of botulinum toxin injection was used in bilateral genioglossus and lateral pterygoid (10 units each) (Maurya et al., 2020). The duration of the treatment was not mentioned in the rest of the studies. Anticholinergic along with botulinum toxin was used in one patient for generalized dystonia and cervical dystonic tremor (Spagnolo et al., 2014). Tracheostomy was done for one patient with laryngeal dystonia (Hamano et al., 2004). Twelve studies did not report on any treatment the patients received.

### 3.6 Outcome

On the basis of dependency for the activities of daily living, the outcome was defined in only four of the studies into poor (bedridden), partial (dependent), and complete (independent) recovery, and the assessment was done at the end of 6 months in three studies, and the end of 3 months in one study (Kalita et al., 2003; Kalita et al., 2011; Maurya et al., 2020; Misra & Kalita, 2010). With this definition, complete recovery was seen in six patients and was poor in four patients from two studies with 22 patients (Kalita et al., 2011; Misra & Kalita, 2002). Dystonia disappeared in 71% of the patients in 6 months among 38 patients in the study by Misra et al. (2010). A significant reduction in dystonia in 3 months' follow‐up from grade 4 to 2 was seen in an 11‐year‐old boy with oromandibular dystonia from Nepal (Maurya et al., 2020). Stridor, as a result of laryngeal dystonia, was improved after tracheostomy in 1 patient (Hamano et al., 2004). Basumatary et al. mentioned that the dystonia improved in all 38 dystonic children (Basumatary et al., 2013). Dystonia in 13 patients had improvement in 6 to 9 months (Dutta et al., 2021). Five patients with dystonic spasms had MSD for a variable period of time ranging from 1 to 6 months and on 6 months' follow‐up of four of these patients, one was ambulatory with support and the rest were bedridden because of fixed axial dystonia, and at the end of 1 year, one patient had a complete recovery, one had a partial recovery, and two were still bedridden (Kalita & Misra, 2000). Eight studies with patients neither defined the outcome nor mentioned the outcome of dystonia, as shown in Table 3.

## 4 DISCUSSION

JE is commonly a disease of children in endemic areas, but in newly affected areas, it infects both adults and children (Misra & Kalita, 2010). Annually about 50,000 cases of JE occur worldwide, and 15,000 of them die. The age‐specific attack rates are highest between 3 and 6 years of age which have been attributed to high outside exposure, especially playing in the evening and subsequent high risk of mosquito bites to poorly clothed children in villages, and the attack rates decline after the age of 14 years owing to high levels of neutralizing antibodies due to natural exposure and subclinical infection (Misra & Kalita, 2010). Children less than 14 years of age comprised 31% of the total JE case as per our review. A study from Cuddalore district, Tamil Nadu, reported 27.3% of the patients were children affected by JE among AES cases that were hospitalized (Kabilan et al., 2004). Our study showed males were more affected by JE as compared to females, with the male to female ratio being 1.78. A study from India showed similar data, with males comprising 58% of all JE cases (Jacobson & Sivalenka, 2004). Almost 89% of the JE cases included in our study were confirmed by JEV‐specific IgM antibody detection in serum and/or CSF, as IgM antibody detection is known to reliably differentiate the JE virus from related flavivirus (Gadkari & Shaikh, 1984).

JE is the most common cause of dystonia among flavivirus (Misra & Kalita, 2010). Our review showed that dystonia was present in 15.13% of the JE patients, with males being more affected corresponding to the male to female ratio in JEV infection. Furthermore, almost 46% of the dystonic patients were children. This could be due to the fact that the age of insult plays an important role in the presence of the type of MD, with dystonia being a much more common finding in patients with younger age of insult (Jhunjhunwala et al., 2014).

Our review showed the development of dystonia was usually reported after 1 to 4 weeks of encephalitis. The period of latency from the insult to the SMD might be due to the time required for inflammation, oxidative reactions, remyelination, ephaptic transmission, trans‐synaptic neuronal degeneration, central synaptic reorganization, and diaschisis mediated by collateral sprouting and denervation supersensitivity (Burke et al., 1980; Hilaire et al., 1991; Jankovic, 1994; Jhunjhunwala et al., 2014). After insult to the brain, dendritic plasticity and changes in the synaptic activity could result in pathological neuronal circuitry that could facilitate the development of MD, including dystonia (Jankovic, 1994; Kalita & Misra, 2000).

Various types of focal dystonia, either alone or in combination, were the most frequent type of dystonia occurring in almost 56% of post‐JE patients, while the remainder had generalized dystonia. Dystonia in JE usually involves both the axial and limb muscle and is commonly of fixed type resulting in opisthotonos, retrocollis, and oromandibular and limb dystonia (Misra, 2017). Dystonia in JE can be very severe from the beginning in some cases, and there can also be worsening of previous mild‐to‐moderate dystonia or precipitation of severe one due to infection or initiation or withdrawal of certain drugs (Kalita & Misra, 2000; Kalita & Misra, 2000). There have been reports of chest or urinary infection leading to worsening of dystonia (Manji, 1998; Vaamonde et al., 1994). Attacks of MSD in five patients from one study included in our review were exacerbated with fever and full bladder while reduced at night. Dystonic spasms can also occur in dystonic patients with fever, tachycardia, exhaustion, perspiration, and breathing and feeding difficulty being the usual results requiring intensive care and sometimes mechanical ventilation (Kalita & Misra, 2000; Kalita & Misra, 2000). Our review highlighted the presence of dystonic spasms in 19 patients with a clinical presentation similar to as mentioned above without the need for any mechanical ventilation. Securing the airway by tracheostomy was done for one patient with laryngeal dystonia having severe respiratory difficulty.

MRI done in JE patients with dystonia revealed abnormal signals in the thalamus, which was the most common site to be involved along with basal ganglia, brainstem, cortex, and substantia nigra. Dystonic patients in whom CT was done showed abnormality in bilateral thalami in all patients, and SPECT revealed perfusion defect and uptake abnormalities. Bilateral T2 hyperintense and T1 isointense to hypointense thalamic lesions, especially hemorrhagic lesions, have been reported as typical findings of JE in a suitable clinical setting (Basumatary et al., 2013).

Damage to numerous regions of the brain can bring out dystonia, most commonly the basal ganglia, but also the thalamus, cerebellum, parietal lobe, and brainstem (Geyer & Bressman, 2006). Dystonia might also develop from partial restraint of competing motor patterns because of insufficient surrounding inhibition of competing motor pattern generators (Mink, 2003). Overflow contraction of the adjacent muscles are led by the declaration of the facilitatory center as a result of defective surrounding inhibition. Improper disinhibition of undesired muscle activity thus occurs due to diminished efficacy of the surrounding area with or without extension of the center (Mink, 2003). Thalamic involvement and perfusion flaw suggest malfunction in thalamocortical and basal ganglia circuits (Kalita et al., 2011; Mink, 2003). Similarly, dysfunction in the cerebello‐thalamo‐cortical pathway and cortico‐striato‐pallido‐thalamo‐cortical pathway along with relative dopamine deficiency or its receptor malfunction can manifest as both generalized and focal dystonia (Perlmutter et al., 1997; Simonyan, 2018). Dystonia in JE patients included in our review can be explained by the involvement of the thalamus, basal ganglia, brainstem, cortex, and substantia nigra. Further, involvement of these areas by JEV leading to MD including dystonia has been supported by the fact that JEV antigen was found distributed in the thalamus, substantia nigra, and medulla oblongata in human autopsies (Desai et al., 1995).

Several classes of medication, including high doses of anticholinergics, gamma‐aminobutyric acid (GABA) agonists, and dopaminergic agents are used in the management of dystonia (Jankovic, 2006). Anticholinergic agents are generally the most successful oral medications, with trihexyphenidyl being the most commonly used agent (Burke et al., 1986; Jankovic, 2006). In a prospective, double‐blind trial of high‐dose trihexyphenidyl, Burke et al. found a clinically significant improvement in 71% of 31 patients (mean age 19 years) on an average daily dose of 30 mg daily during a 36‐week study period (Burke et al., 1986). With the oral medications most frequently used for dystonic patients in the studies included in our review, trihexyphenidyl was used in all oral medication combinations. Despite the lack of evidence for efficacy in absence of large controlled studies, benzodiazepines are often used in dystonia, clonazepam being the most commonly used (Fahn, 1983). In an open study, clonazepam and other benzodiazepines were found to be beneficial in 16% of patients with various types of dystonia (Fahn, 1983). TBZ depletes vesicular stores of dopamine by inhibiting the vesicular monoamine transporter 2 (VMAT‐2) and is another effective drug for the treatment of dystonia as well as other hyperkinetic MD, such as chorea, tics, tardive dyskinesia, and myoclonus (Kenney et al., 2007). Clonazepam and TBZ were also used for the treatment of dystonia in the patients included in our study along with other oral medications.

Intramuscular injections of botulinum toxin can reduce symptoms of focal as well as generalized dystonia in some cases by acting at the neuromuscular junction (Dressler & Adib Saberi, 2005). Botulinum toxin, a toxic protein produced by the bacterium *Clostridium botulinum*, exerts its therapeutic benefit by blocking the release of acetylcholine into the neuromuscular junction and thus, reduces the excessive activity of affected muscles in dystonia (Breakefield et al., 2008; Dressler & Adib Saberi, 2005). A patient with oromandibular dystonia was given oral medication for 2 weeks, and when no improvement was seen, botulinum toxin was then used, which caused a significant reduction in dystonia from grade 4 to grade 2. Botulinum toxin has become the treatment of choice for most patients with focal or segmental dystonia, including those with blepharospasm, spasmodic dysphonia, and cervical, oromandibular, and lingual dystonia (Cloud & Jinnah, 2010).

Apart from the oral drugs mentioned above, numerous other oral pharmaceutical agents either alone or in combinations have been mentioned in anecdotal reports in the improvement of dystonia, which include pregabalin, midazolam, pimozide, eperisone, verapamil, gabapentin, levetiracetam, and vitamin E (van den Heuvel et al., 2016). Without the availability of strong evidence in support of the use of any oral drugs for acquired dystonia, the location of dystonia has no bearing on the choice of medication, and among the various drugs available, usually anticholinergic is started as a first‐line and baclofen or clonazepam as a second‐line agent (Termsarasab et al., 2016; van den Heuvel et al., 2016).

Although no patient in our study underwent surgical procedures for acquired dystonia secondary to JE, these can be done if the above‐mentioned treatment modalities fail or provide inadequate relief. Surgical denervation, continuous intrathecal (ITB) or intraventricular (IVB) baclofen therapy, stereotactic lesioning, and neurostimulation, including motor cortex stimulation (MCS) and deep brain stimulation (DBS), are among several surgical interventions that have been performed so far (van den Heuvel et al., 2016).

Drug treatment for MSD is often empirical due to inadequate knowledge about the pharmacological basis of MSD (Marsden et al., 1984). Once levodopa responsive dystonia is excluded, anticholinergics, haloperidol, and TBZ and its analogs are used (Marsden et al., 1984). Markedly severe dystonic patients included in our review were first given oral medications in combinations, and they eventually improved over 6 months; only one patient was prescribed botulinum toxin injection on top of oral drugs for grade 4 dystonia leading to a downgrading of grade to 2 in 3 months' follow‐up. Some anecdotal reports also recommend using anticonvulsants and baclofen (Vaamonde et al., 1994). Muscle relaxants, along with sedatives, might prove useful in controlling painful spasms of dystonia (Kalita & Misra, 2000; Vaamonde et al., 1994). Haloperidol and diazepam were used in patients with severe dystonic spasms as mentioned in our review.

Dystonia in the patients post‐JE included in our review has regressed over a variable amount of time during follow‐up. This improvement in dystonia secondary to JE is consistent with the natural history of encephalitis in which there is a regressive course for most MD, and even complete recovery is seen in milder ones (Kalita et al., 2003; Misra & Kalita, 2002). When compared with other MD following JE, like parkinsonism, dystonia regressed at a slower rate during the follow‐up period, and the presence of dystonia might suggest more severe illness and a poorer prognosis (Misra & Kalita, 2002).

### 4.1 Limitations

The major strength of our study is that this is the first systematic review conducted exploring dystonia post‐JE which is a major cause of encephalitis. However, our review has several limitations. This study has covered data from only seven countries, which is the main shortcoming. The exclusion of non‐English articles was another limitation. Unpublished literature was not included, and we did not contact authors for unpublished supplementary data as well. Due to the lack of enough studies, detailed information in botulinum toxin and surgical management of dystonia could not be explored in depth.

## 5 CONCLUSION

Dystonia can be a disabling sequela of JE infection. Along with supportive measures for JE infection, symptomatic medical therapy can significantly improve the quality of life and should not be overlooked. There is still a lack of studies focusing on the assessment of various MDs associated with JE. Further longitudinal studies including a large number of patients will be needed to be clear about the clinical course, treatment, and outcome of dystonia.

## FUNDING INFORMATION

No funding was received.

## CONFLICT OF INTEREST

None of the authors has any conflict of interest to disclose. We confirm that we have read the Journal's position on issues involved in ethical publication and affirm that this report is consistent with those guidelines.

## AUTHOR CONTRIBUTIONS

RA, SS, and SH conceptualized and designed the study. RA, SS, and SH were involved in collecting and analyzing data, and writing the manuscript. SK, SC, KS, RK, RR, BPG, and RO were involved in revising the manuscript critically for important intellectual content. RO supervised the research and manuscript writing process. All authors were involved in the review of the manuscript. Furthermore, all the authors read and approved the final version of the manuscript.

### TRANSPARENT PEER REVIEW

The peer review history for this article is available at https://publons.com/publon/10.1002/brb3.2496

## Supporting information

SUPPORTING INFORMATION

## Data Availability

The datasets of the current study are available from the corresponding author on reasonable request.
