# Supplementary material for: Clinical spectrum and management of dystonia in patients with Japanese encephalitis: A systematic review
Source: Brain Behav. 2022 Jan 13;12(2):e2496. doi: 10.1002/brb3.2496 (PMC8865161; doi:10.1002/brb3.2496)
Supplement: Supplementary file 1 — SUPPORTING INFORMATION [file BRB3-12-e2496-s001.docx]

**Supplementary Content**

**Appendix 1:** Search strategy used in the current systematic review

This supplementary material has been provided by the authors to give readers additional information.

**Appendix 1:** Search strategy used in the current systematic review.

PubMed Search Strategy:

(((((dystonia) OR (dystonic disorder)) OR (movement disorder)) OR (dyskinesia)) OR (muscle tonus)) OR (muscle dystonia) 250,738

(((((japanese encephalitis) OR (JE)) OR (Japanese B Viral Encephalitis)) OR (Viral Encephalitis, Japanese B)) OR (japanese B encephalitis)) OR (viral encephalitis) 49,597

(((((dystonia) OR (dystonic disorder)) OR (movement disorder)) OR (dyskinesia)) OR (muscle tonus)) OR (muscle dystonia)) AND ((((((japanese encephalitis) OR (JE)) OR (Japanese B Viral Encephalitis)) OR (Viral Encephalitis, Japanese B)) OR (japanese B encephalitis)) OR (viral encephalitis)) Filters: from 2000 - 2021 580

Embase Search Strategy:

#1: ('dystonia'/exp OR 'dystonia' OR 'dystonic disorder'/exp OR 'dystonic disorder' OR 'motor dysfunction'/exp OR 'motor dysfunction') AND [2000-2021]/py

#2: 'japanese encephalitis virus group' OR 'japanese encephalitis virus' OR 'japanese encephalitis' OR 'virus encephalitis'

#3: #1 AND #2

#3. (('dystonia'/exp OR 'dystonia' OR 'dystonic 1,403

disorder'/exp OR 'dystonic disorder' OR 'motor

dysfunction'/exp OR 'motor dysfunction') AND

[2000-2021]/py) AND ('japanese encephalitis virus

group' OR 'japanese encephalitis virus' OR

'japanese encephalitis' OR 'virus encephalitis')

#2. 'japanese encephalitis virus group' OR 'japanese 15,264

encephalitis virus' OR 'japanese encephalitis' OR

'virus encephalitis'

#1. ('dystonia'/exp OR 'dystonia' OR 'dystonic 656,627

disorder'/exp OR 'dystonic disorder' OR 'motor

dysfunction'/exp OR 'motor dysfunction') AND

[2000-2021]/py
